# Sprouty2 limits intestinal tuft and goblet cell numbers through GSK3β-mediated restriction of epithelial IL-33

**DOI:** 10.1038/s41467-021-21113-7

**Published:** 2021-02-05

**Authors:** Michael A. Schumacher, Jonathan J. Hsieh, Cambrian Y. Liu, Keren L. Appel, Amanda Waddell, Dana Almohazey, Kay Katada, Jessica K. Bernard, Edie B. Bucar, Safina Gadeock, Kathryn M. Maselli, M. Kay Washington, Tracy C. Grikscheit, David Warburton, Michael J. Rosen, Mark R. Frey

**Affiliations:** 1 The Saban Research Institute, Children’s Hospital Los Angeles, Los Angeles, CA USA; 2 Department of Pediatrics and Department of Biochemistry and Molecular Medicine, University of Southern California Keck School of Medicine, Los Angeles, CA USA; 3 Division of Gastroenterology, Hepatology and Nutrition, Cincinnati Children’s Hospital Medical Center, Cincinnati, OH USA; 4 Department of Stem Cell Research, Institute for Research and Medical Consultation (IRMC), Imam Abdulrahman Bin Faisal University, Dammam, Saudi Arabia; 5 Departments of Pathology, Microbiology, and Immunology, Vanderbilt Ingram Cancer Center, Vanderbilt University Medical Center, Nashville, TN USA; 6 Department of Surgery, Keck School of Medicine, University of Southern California, Los Angeles, CA USA; 7 Department of Pediatrics, University of Cincinnati College of Medicine, Cincinnati, OH USA

**Keywords:** Growth factor signalling, Differentiation, Interleukins, Inflammatory bowel disease

## Abstract

Dynamic regulation of intestinal cell differentiation is crucial for both homeostasis and the response to injury or inflammation. Sprouty2, an intracellular signaling regulator, controls pathways including PI3K and MAPKs that are implicated in differentiation and are dysregulated in inflammatory bowel disease. Here, we ask whether Sprouty2 controls secretory cell differentiation and the response to colitis. We report that colonic epithelial Sprouty2 deletion leads to expanded tuft and goblet cell populations. Sprouty2 loss induces PI3K/Akt signaling, leading to GSK3β inhibition and epithelial interleukin (IL)-33 expression. In vivo, this results in increased stromal IL-13+ cells. IL-13 in turn induces tuft and goblet cell expansion in vitro and in vivo. Sprouty2 is downregulated by acute inflammation; this appears to be a protective response, as VillinCre;Sprouty2^F/F^ mice are resistant to DSS colitis. In contrast, Sprouty2 is elevated in chronic colitis and in colons of inflammatory bowel disease patients, suggesting that this protective epithelial-stromal signaling mechanism is lost in disease.

## Abstract (web summary)

Dynamic regulation of colonic secretory cell numbers is a critical component of the response to intestinal injury and inflammation. Here, the authors show that loss of the intracellular signalling regulator Sprouty2 in the intestinal epithelial cells is a protective response to injury that leads to increased secretory cell numbers, thus limiting colitis severity.

## Introduction

Tight control of secretory cell differentiation is crucial for maintaining a healthy intestinal epithelium. For example, secretory tuft and goblet cells perform defensive roles against disease by supporting tissue maintenance and repair and providing a physical mucus barrier against the external environment^1–3^, and defects in these cells predispose to or exacerbate barrier dysfunction and inflammation^4,5^. On the other hand, excessive secretory cell development at the expense of absorptive lineages could in theory also be detrimental. Like many elements of intestinal physiology, a careful balance in a Goldilocks zone is required. Furthermore, the optimal mix of lineages may change rapidly in the face of injury or inflammation^6–8^, requiring the organ to rapidly alter its cellular composition to deal with current conditions.

Despite the importance of tight, and apparently dynamic, regulation of secretory differentiation, relatively little is known about how this balance is maintained. What is clear is that intracellular signaling plays a major role. PI3K and MAPK signals rapidly alter the balance of secretory cell development^9–11^. Some of these pathways are dysregulated in inflammatory bowel disease (IBD), and defects in them likely play a key role in epithelial dysfunction during chronic inflammation^12,13^. However, the mechanisms by which diverse signals (MAPKs, PI3K/Akt, Wnts, etc.) are integrated and regulated in the gut are not well understood.

Sprouty2 is a widely expressed regulator and integrator of intracellular signaling downstream of receptor tyrosine kinases. It was originally identified as a critical player in branching morphogenesis in the developing mammalian lung^14–17^, and its ability to bind and inhibit Raf^18,19^ placed it as a regulator of the canonical MAPK cascade (i.e., Ras-Raf-MEK-ERK). Since then, docking sites on Sprouty2 for molecules such as the ubiquitin ligase Cbl^20,21^, the adapter protein Grb2 (ref. ^22^), Src^22,23^, and components of the PI3K pathway^24,25^ have been identified, pointing to a broader and more integrative role in controlling and shaping signaling. As these pathways are involved in the cellular differentiation and immune responses in various tissue types, we sought to determine the function of Sprouty2 in the colonic epithelium.

To address this question, we used coordinated in vivo (intestinal epithelial-specific Sprouty2 knockout mice) and in vitro (colonoid culture) approaches to study how Sprouty2 fine-tunes the intestinal environment by modulating epithelial function, cell differentiation, and cytokine production. We find that Sprouty2 is a key regulator of tuft and goblet cell census in the colon, through an epithelial–stromal circuit involving PI3K, GSK3β, IL-33, and IL-13. Furthermore, Sprouty2 expression was responsive to acute inflammatory stimuli, suggesting that it is a mediator of dynamic changes to secretory cell populations in response to acute challenge. While inflammatory cytokine-induced suppression of Sprouty2 was conserved in healthy human tissues, Sprouty2 levels were in contrast elevated in human IBD. Thus, loss of this mechanism potentially contributes to disease by removing a compensatory response to injury and inflammation.

## Results

### Sprouty2 is robustly expressed in the colonic epithelium but is repressed by inflammation

**Fig. 1:**
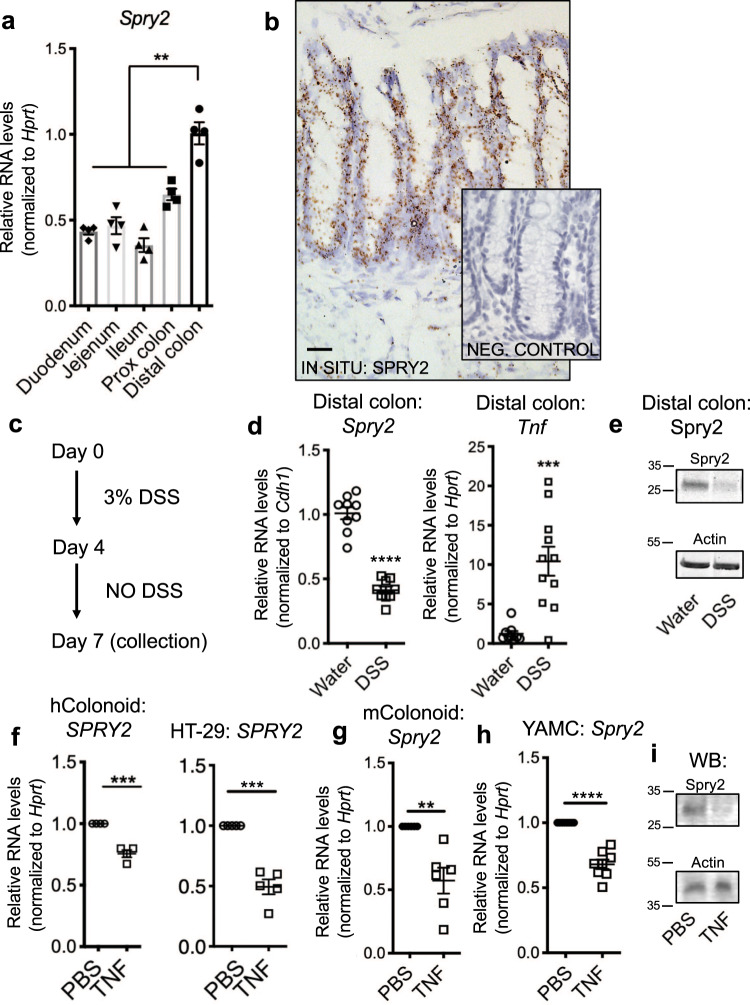
Sprouty2 is highly expressed in the colonic epithelium and repressed by inflammation. **a** Expression of *Spry2* was analyzed in tissue homogenates along the length of the intestinal tract by qPCR (*n* = 4 mice; **p < 0.01). **b** In situ analysis of murine distal colonic sections showed *Spry2* is predominantly expressed within the epithelial compartment. Scale bar = 25 µm; representative of stains of three mice. **c** Mice were subjected to 3% DSS in drinking water for 4 days followed by 3 days chase to induce colitis and **d** distal colonic homogenates were analyzed by qPCR for *Sprouty2* (*n* = 9 mice per group; ****p < 0.0001) and *Tnf* (*n* = 11 mice per group, ***p = 0.0003). **e** Western blot for Sprouty2 on colonic homogenate from water and DSS-treated mice (representative of four mice per group). **f** Primary human (*n* = 4 independent cultures derived from four surgical specimens; ***p = 0.0002) colonic epithelial colonoids, human colonic cells (HT-29; *n* = 5 independent experiments; ***p = 0.0003), **g** murine (*n* = 6 cultures derived from six mice; **p = 0.0018) colonoids, and **h** immortalized mouse colon cells (YAMC; *n* = 8 independent experiments; ****p < 0.0001) were treated with TNF (100 ng/ml) for 24 h and analyzed by qPCR for *SPRY2* levels. **i** Western blot for Sprouty2 in TNF-treated YAMC cells after 24 h (representative of four independent experiments). Data in this figure are presented as mean ± SEM. Analyzed by **a** one-way ANOVA with Tukey post hoc test or **d**, **f**–**h** two-sided *t* test.

As a first step toward identifying the function of Sprouty2 in the gut, we analyzed its expression pattern. *Spry2* was present throughout the intestinal tract, with highest levels in the colon (Fig. 1a). It was enriched in the epithelium, with low expression in the subepithelial space (Fig. 1b). As an integrator of extracellular stimuli, Sprouty2 expression is repressed by inflammatory challenge in airway epithelial cells^26^; to test if it is similarly regulated in the colon, we subjected mice to dextran sodium sulfate (DSS) colitis for 4 days to cause acute damage, followed by 3 days of recovery (Fig. 1c). Sprouty2 RNA and protein levels were significantly reduced by 3 days post DSS injury (Fig. 1d, e), correlating with elevated *Tnf* (Fig. 1d). In vitro, TNF inhibited *SPRY2* expression in human colonic epithelial organoid cultures (colonoids), cultured human (HT-29) colonocytes (Fig. 1f), murine colonoids (Fig. 1g), and cultured mouse (YAMC) colonocytes (Fig. 1h, i). Collectively, these data show that Sprouty2 is an inflammation-regulated epithelial target both in vivo and in vitro.

### Tuft and goblet cell numbers are increased in the colons of Spry2^IEKO^ mice

**Fig. 2:**
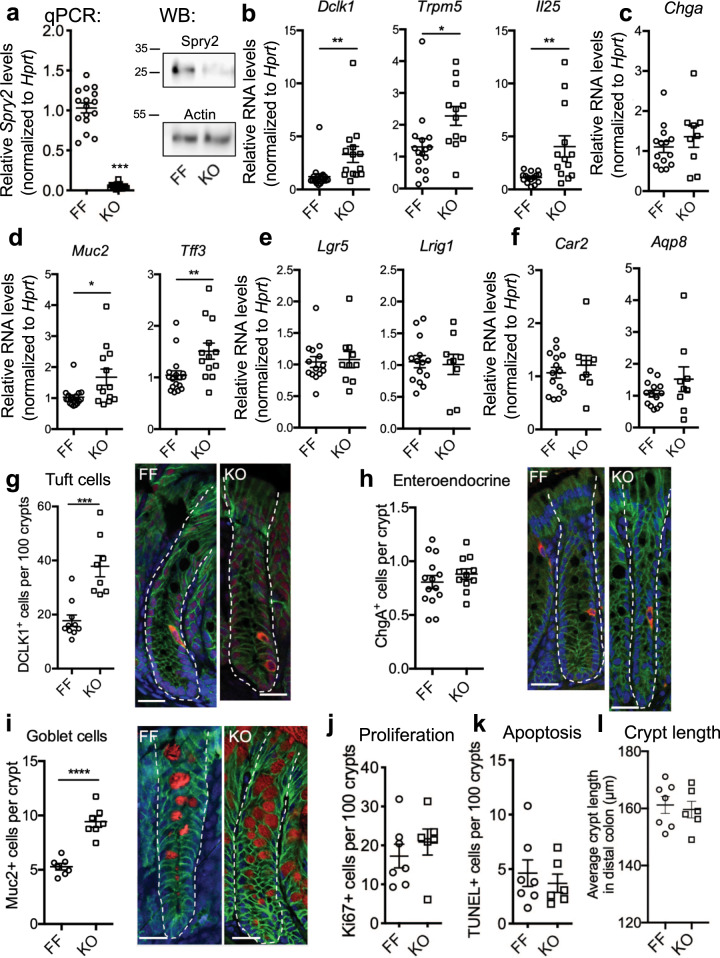
Loss of epithelial Sprouty2 results in expansion of colonic tuft and goblet cell numbers. **a** Deletion of Sprouty2 in Spry2^IEKO^ mice was confirmed by qPCR and western blot of colonic homogenates. **b** Tuft (*Dclk1*, *Trpm5*, and *Il25*), **c** enteroendocrine (*Chga*), **d** goblet (*Muc2* and *Tff3*), **e** stem (*Lgr5* and *Lrig1*), and **f** absorptive enterocyte (*Car2* and *Aqp8*) markers were measured in Spry2^IEKO^ colonic homogenates by qPCR. **g** Tuft (Dclk1^+^; in red), **h** enteroendocrine (ChgA^+^; in red), and **i** goblet (Muc2^+^; in red) cells were quantified by immunofluorescence staining of distal colonic sections. Green = E-cadherin. Scale bar = 25 µm. **j** Proliferation (Ki67+), **k** apoptosis (TUNEL+), and **l** crypt length were quantified in distal colons. Data are presented as mean ± SEM. Analyzed by two-sided *t* test. *p < 0.05; **p < 0.01; ***p < 0.001; ****p < 0.0001. Exact *p* values where appropriate are as follows **b** *Dclk1 p* = 0.0065; *Trpm5 p* = 0.0203; *Il25 p* = 0.0046; **d** *Muc2 p* = 0.0124; *Tff3 p* = 0.0099; **g** *p* = 0.0002. Number of mice analyzed, from five independent litters, in each panel as follows: **a** 16 per group; **b** *Dclk1* 19 FF, 14 KO; *Trpm5* 16 FF, 12 KO; *Il25* 15 FF, 13 KO; **c** 14 FF, 9 KO; **d** *Muc2* 17 FF, 13 KO; *Tff3* 17 FF, 13 KO; **e**, **f** 14 FF, 9 KO; **g** 10 FF, 8 KO; **h** 14 FF, 8 KO; **i** 7 FF, 7 KO; and **j**–**l** 7 FF, 6 KO.

To understand the functional outcome of inflammation-induced Sprouty2 repression in the colonic epithelium, we generated intestinal epithelial-specific Sprouty2 deletion mice (VillinCre;Spry2^flox/flox^, hereafter termed Spry2^IEKO^; Fig. 2a). Since the MAPK and PI3K cascades regulated by Sprouty2 are intimately involved in differentiation^9,11^, we analyzed lineage markers (Fig. 2b–f) and found a significant increase in tuft (*Dclk1*, *Trpm5*, and *Il25*) and goblet (*Muc2* and *Tff3*) cell marker expression in colonic homogenates of Spry2^IEKO^ mice compared to Cre-negative littermate controls. In contrast, markers for enteroendocrine (*ChgA*), stem (*Lgr5*, *Lrig1*), and absorptive (*Car2, Aqp8)* cells did not change, suggesting specificity in the response. To confirm that RNA changes correlated with increased tuft and goblet cell numbers, we stained colonic sections for Dclk1, chromogranin A, and Muc2 (Fig. 2g–i). Spry2^IEKO^ mice showed an increase in the number of both tuft and goblet cells, but not enteroendocrine cells. The differential regulation of secretory cells suggests an additional modulator of secretory pathways may be required for enteroendocrine specification^27^. To test whether *Spry2* deletion altered the epithelial cell life cycle, we analyzed proliferation and apoptosis in distal colon, but found no differences between Spry2^IEKO^ mice and control littermates (Fig. 2j, k). We also observed no differences in crypt length (Fig. 2l).

### Sprouty2 deletion induces colonic epithelial IL-33 expression

**Fig. 3:**
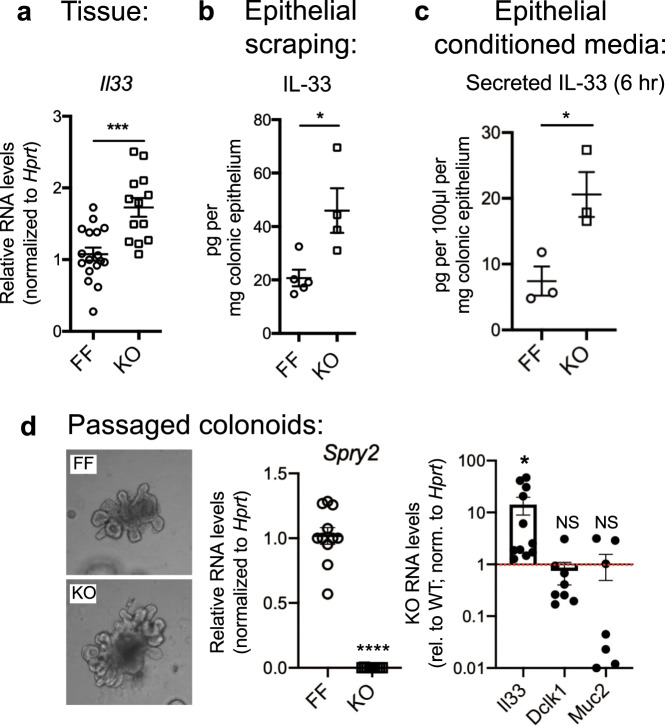
Colonic epithelial IL-33 expression is increased in Spry2^IEKO^ mice. **a** *Il33* expression was measured by qPCR in colonic homogenates from Spry2^FF^ and Spry2^IEKO^ mice (*n* = 17 FF, 13 KO; ***p = 0.0002). **b** IL-33 protein was measured in colonic epithelial scrapings by ELISA (*n* = 5 FF, 4 KO; *p = 0.0172). **c** Secreted IL-33 was measured in conditioned media from epithelial peels after 6 h incubation by ELISA (*n* = 3 per group; *p = 0.0314). **d** Expression of *Spry2* (*n* = 11 FF, 9 KO cultures; ****p < 0.0001), *Il33* (*n* = 11 cultures; *p = 0.0221)*, Dclk1* (*n* = 8 cultures), and *Muc2* (*n* = 7 cultures) was assayed by qPCR in passaged colonoids generated from Spry2^FF^ and Spry2^IEKO^ mice. *Il33*, *Dclk1*, and *Muc2* expression in Spry2^IEKO^ is presented relative to Spry2^FF^ mice (indicated by red dashed line *n* = 11 cultures). In figure, each colonoid culture was generated from a separate mouse and data are presented as mean ± SEM. Analyzed by two-sided *t* test.

The intestinal epithelium is a potent producer of cytokines that shape epithelial and immune responses in the gut. IL-33 is a member of the IL-1 superfamily with reported expression in both the epithelium and mesenchyme. It is linked to secretory cell development, which may be a response mechanism to protect the epithelium from insult. However, the environmental and intracellular signals differentially regulating IL-33 expression in different cell types are not well understood. To test whether Sprouty2 might influence tuft and goblet cell expression through modulation of IL-33 expression, we first performed qPCR analysis on distal colonic homogenates. We observed a significant increase in *Il33* expression in Spry2^IEKO^ mice versus control littermates (Fig. 3a). IL-33 induction specifically in the epithelium was confirmed by ELISA on distal colonic epithelial scrapings (Fig. 3b). To test for IL-33 release, we cultured isolated distal colonic epithelia for 6 h. Conditioned media from these cultures showed elevated IL-33 release from Sprouty2-null cultures (Fig. 3c). Similar to the in vivo results, *Il33* was durably elevated in passaged knockout colonoids (Fig. 3d), specifically demonstrating increased epithelial expression. Interestingly, while tuft and goblet cell markers were elevated in Sprouty2-null epithelia in vivo (Fig. 2), this was not the case in passaged colonoids (Fig. 3d), suggesting a requirement for an additional, extra-epithelial signal.

### Tuft and goblet cell expansion in Spry2^IEKO^ mice requires stromal signals

**Fig. 4:**
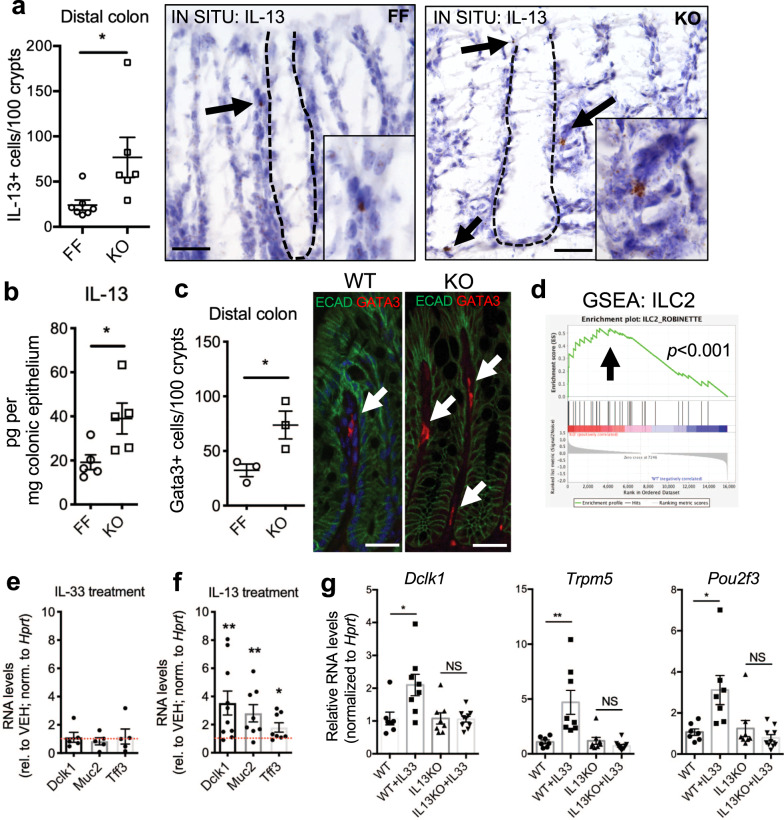
IL-33-driven tuft and goblet cell expansion requires stromal IL-13. **a** Distal colonic sections from Spry2^FF^ and Spry2^IEKO^ mice were probed for *Il13* by in situ RNAscope analysis and counted for IL-13^+^ cells per 100 crypts (dashed line around colonic crypt at base of epithelium); *n* = 7 FF, 6 KO mice; *p = 0.0301. Scale bar = 25 µm. **b** Epithelial scrapings from Spry2^FF^ and Spry2^IEKO^ mice were measured for IL-13 levels by ELISA; *n* = 5 per group; *p = 0.0338. **c** Number of Gata3^+^ cells was quantified in distal colonic tissue. Gata3 = red, E-cadherin = green; *n* = 3 per group; *p = 0.0409. Scale bar = 25 µm. **d** Gene set enrichment analysis (GSEA) using a published intestinal ILC2 gene signature from bulk tissue^31^ was performed on RNA sequencing of distal colonic homogenates; *n* = 6 per group. Colonoids generated from WT mice were treated with **e** IL-33 (100 ng/ml, *n* = 5 cultures per group) or **f** IL-13 (10 ng/ml, *n* = 8 cultures per group) for 24 h, and analyzed for tuft (*Dclk1*) and goblet cell (*Muc2, Tff3*) marker expression. Each colonoid culture was from a different mouse. *p < 0.05; **p < 0.01. **g** WT and IL-13^*−/−*^ mice were i.p. injected with PBS or IL-33 for 4 days, and distal colonic expression of tuft cell markers (*Dclk1*, *Trpm5*, and *Pou2f3*) was assayed by qPCR, revealing a requirement for IL-13 in IL-33 mediated tuft cell induction in the colon; *n* = 7 WT + PBS, 8 WT + IL-33, 8 IL-13KO + PBS, and 10 IL-13KO + IL-33. *p < 0.05; **p < 0.01. Data are presented as mean ± SEM. Analyzed by **a**–**f** two-sided *t* test or **g** one-way ANOVA with Tukey post hoc test.

In the intestine^1,6,28^, stomach^29^, and skin^30^ IL-33 can drive IL-13 release from type 2 innate lymphoid cells (ILC2s). We performed in situ analysis for *Il13*^+^ cells and found a significant increase in their numbers within colonic stromal tissue of Spry2^IEKO^ mice (Fig. 4a). We also found elevated levels of IL-13 protein in distal colonic epithelial scrapings by ELISA (Fig. 4b). To further confirm ILC2 numbers, we stained sections for Gata3, a marker expressed by ILC2s^31,32^ and observed elevated numbers of Gata3^+^ cells within the colonic epithelium. Furthermore, we detected enrichment of an intestinal ILC2 signature^31^ by gene set enrichment analysis on RNA-sequencing results from colons of these mice (Fig. 4d).

To test if tuft and goblet cell expansion is an epithelial-intrinsic response to IL-33 or in contrast requires IL-13, we treated colonoids from wild-type mice with these cytokines. Exogenous IL-33 was not sufficient to promote expression of tuft *(Dclk1)* or goblet *(Muc2* and *Tff3)* cell markers in colonoids (Fig. 4e), while IL-13 robustly induced these genes (Fig. 4f). In vivo, we found that tuft cell marker induction in the colon by IL-33 injection did not occur in IL-13 KO mice (Fig. 4g), extending previous work demonstrating similar findings for goblet cells^33^. Together these results suggest an epithelial–stromal circuit in which Sprouty2 downregulation allows for IL-33 expression in the epithelium, promoting IL-13 release in the subepithelial cells which in turn signals back to the epithelium to alter cell differentiation patterns, consistent with a recent study on IL-33-driven ileal goblet cell hyperplasia^33^.

**Fig. 5:**
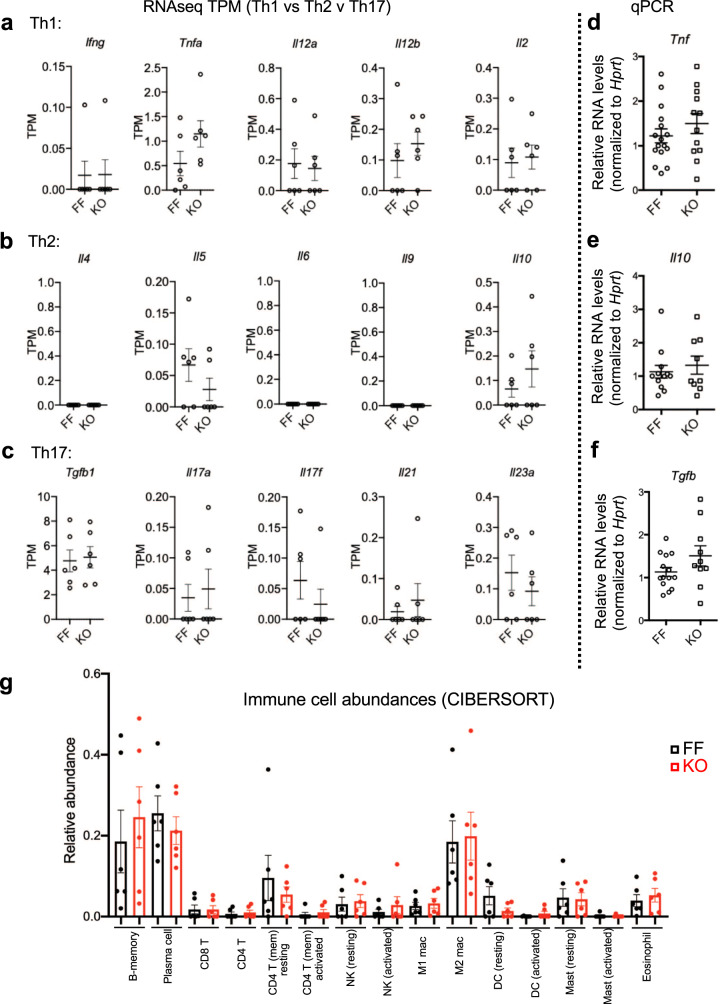
Canonical markers of Th1, Th2, and Th17 immunity are unchanged in Spry2^IEKO^ mice. RNA sequencing was performed on distal colonic homogenates from Spry2^FF^ and Spry2^IEKO^ mice. **a**–**c** Expression levels (as transcripts per million, TPM) of canonical Th1, Th2, and Th17 cytokines were compared between groups; *n* = 6 mice per group. **d**–**f** Expression of principal drivers of Th1, Th2, and Th17 immunity were confirmed by qPCR in a larger cohort of mice (*Tnf*, *n* = 16 FF, 12 KO; *Il10*; *n* = 12 FF, 9 KO; and *Tgfb*, *n* = 14 FF, 10 KO). **g** The relative abundance of immune cell subtypes was estimated in distal colons of Spry2^FF^ and Spry2^IEKO^ mice by CIBERSORT; *n* = 6 mice per group. Data are presented as mean ± SEM. Analyzed by two-sided *t* test.

Increased ILC2 presence is frequently associated with activation of Th2 immunity (e.g., in response to Helminth infection). To test whether loss of epithelial Sprouty2 drives substantial changes in the baseline immune profile of the colon, we examined canonical cytokine markers for Th1, Th2, or Th17 immunity in RNA-sequencing data generated from distal colonic homogenates of Spry2^FF^ and Spry2^IEKO^ mice. We detected no significant differences between genotypes, suggesting the baseline immune profile is not highly altered by loss of Sprouty2 (Fig. 5a–c). These findings were confirmed by qPCR analysis in a subset of cytokine markers (Fig. 5d–f). As an ancillary approach to identifying changes in the immune cell landscape, we next used a computational approach to identifying specific changes in immune cell subsets within the colon. We applied the well-validated CIBERSORT algorithm^34^ to our dataset. This computational cell-type identification method estimates relative immune cell abundances from a mixed cell input, and has been previously validated for use in intestinal tissues^35,36^. Results from this analysis indicated no significant differences in relative abundance of major leukocyte and lymphocyte subtypes detectable with the immune gene set^37^ applied (Fig. 5g), suggesting loss of Sprouty2 does not broadly drive changes in the colonic immune profile, but is rather specific to ILC2s.

### Sprouty2 loss leads to GSK3β inhibition, thus promoting colonic IL-33 expression

**Fig. 6:**
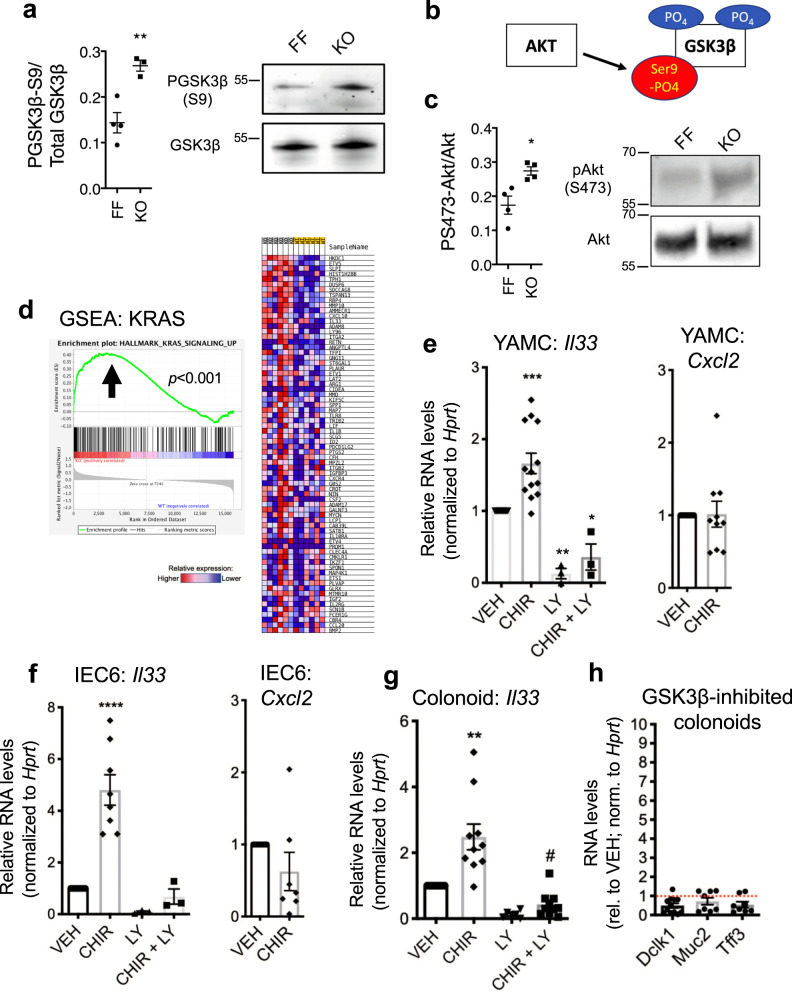
Sprouty2 loss leads to GSK3β inhibition, thus promoting colonic IL-33 expression. **a** Serine 9 phospho-GSK3β levels (**p = 0.0068) were measured in colonic homogenates from Spry2^FF^ (FF) and Spry2^IEKO^ mice (KO) by western blot. **b** Schematic of the Akt inhibitory phosphorylation of serine 9 on GSK3β. **c** Serine-473 phospho-Akt levels (*p = 0.0141) were measured in colonic homogenates from Spry2^FF^ (FF) and Spry2^IEKO^ mice (KO) by western blot. **d** Gene set enrichment analysis (GSEA) using the hallmark K-Ras gene signature was performed on RNA-sequencing results from distal colonic homogenates. Gene expression is defined in key where red indicates relative higher levels and blue indicates relative lower levels, per rank list metric in enrichment plot. **e**, **f** The effects of GSK3β inhibition (using CHIR99021, 3 µM) and/or PI3K/Akt inhibition (using LY294002, 10 µM) for 24 h on *Il33* and *Cxcl2* expression were measured in mouse colon (YAMC) cells, and rat intestinal epithelial (IEC-6) cells by qPCR. *p < 0.05; **p < 0.01; ***p < 0.001; ****p < 0.0001. **g** *Il33* levels were measured in GSK3β or PI3K/Akt inhibited murine colonoids by qPCR. **p < 0.01 versus VEH; #*p* < 0.05 versus LY. **h** *Dclk1*, *Muc2*, and *Tff3* were measured in GSK3β inhibited murine colonoids by qPCR (presented as expression relative to vehicle treated group, indicated by dashed red line). In figure, each colonoid culture was generated from a separate mouse. Data are presented as mean ± SEM. Analyzed by one-way ANOVA with Tukey post hoc test or two-sided *t* test. Number of independent biological replicates for each dataset is: **a** *n* = 4 FF, 3 KO; **c** *n* = 4 per group; **d** *n* = 6 per group; **e** 13 independent experiments for *Il33*, VEH versus CHIR; 3 independent experiments for LY versus CHIR + LY; and for *Cxcl2*, VEH versus CHIR, *n* = 10 independent experiments. **f** Eight independent experiments for *Il33*, VEH versus CHIR; three independent experiments for LY versus CHIR + LY; for *Cxcl2*, seven independent experiments. In **g**, independent cultures for *Il33*, VEH, *n* = 10; CHIR, *n* = 10; LY, *n* = 6; and CHIR + LY, *n* = 10. In **h**, *Dclk*, *n* = 12 cultures; *Muc2*, *n* = 8 cultures; and Tff3, *n* = 8 cultures, (all compared to VEH *n* = 10).

To understand the precise signaling mechanisms through which Sprouty2 controls epithelial IL-33, we performed a screen for phosphorylated growth factor receptor signaling outputs on Spry2^IEKO^ and Cre-negative Spry2^FF^ littermates. This analysis showed increased serine 9 phosphorylation on the Wnt regulator, GSK3β, in Spry2^IEKO^ mucosa, shown here by western blot on distal colonic lysates demonstrating elevated S9 GSK3β phosphorylation in Sprouty2-null colon (Fig. 6a). S9 is an Akt-mediated inhibitory phosphosite on GSK3β^38^ (see schematic in Fig. 6b), and western blot analysis for Ser473 phospho-Akt identified a significant increase in Spry2^IEKO^ mice (Fig. 6c). Furthermore, hallmark gene set enrichment analysis on the RNA-sequencing data from distal colon showed elevated K-Ras signaling (which includes PI3K and PI3K targets) in Spry2^IEKO^ mice (Fig. 6d). As GSK3β can regulate cytokine levels in some tissues, we asked whether it controls IL-33 expression in the colon and intestine. In both mouse colon epithelial cells (YAMC) and rat intestinal epithelial cells (IEC-6), inhibition of GSK3β using the specific inhibitor CHIR99021 resulted in increased *Il33* levels (Fig. 6e, f). Levels of the epithelial-expressed cytokine *Cxcl2* were unaltered, demonstrating this response was not a generalized cytokine regulation (Fig. 6e, f). Next, since Akt is negatively regulated by Sprouty2 (ref. ^24^) and is responsible for serine 9 phosphorylation on GSK3β^38^, we also hypothesized that Sprouty2 loss-driven *Il33* induction is through PI3K/Akt. In YAMC and IEC-6 cells, *Il33* levels were substantially reduced by PI3K/Akt inhibition (Fig. 6e, f). The same findings on *Il33* were observed in primary colon epithelial culture using colonoids (Fig. 6g). Like IL-33 treatment, GSK3β inhibition was also unable to induce tuft and goblet cell markers in colonoids (Fig. 6h). Together these data suggest that Sprouty2 repression of PI3K/Akt signaling is permissive for GSK3β activity, and that a baseline inhibition of IL-33 is released when Sprouty2 is repressed by inflammation.

### Spry2^IEKO^ mice are protected against DSS colitis

**Fig. 7:**
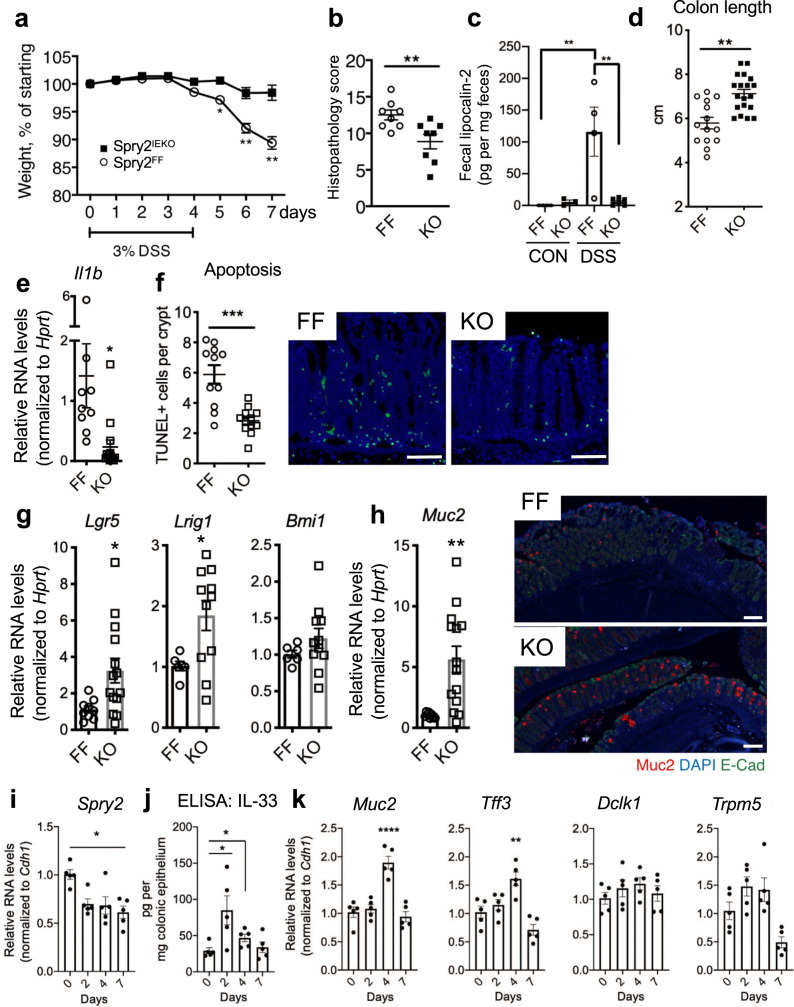
Spry2^IEKO^ mice are protected against DSS colitis. Spry2^FF^ and Spry2^IEKO^ mice were given 3% DSS in drinking water for 4 days, followed by 3 days of plain drinking water to induce colitis. **a** Weights were measured daily; *p < 0.05; **p < 0.01. **b** Colonic sections were scored by a blinded pathologist; **p = 0.0099. **c** Fecal lipocalin-2, a marker of intestinal inflammation, was measured by ELISA; **p < 0.01. **d** Colon lengths were measured (*p* = 0.0002) and **e** expression of *Il1b* was assayed in distal colonic homogenates by qPCR between DSS-treated groups at day 7 (*p* = 0.0146). **f** Apoptosis rate in distal colon (*p* = 0.0001) was measured by TUNEL stain (green). Scale bar = 75 µm. Expression of **g** *Lgr5* (*p* = 0.0212), *Lrig1* (*p* = 0.0280), *Bmi1*, and **h** *Muc2* (*p* = 0.0028) were analyzed by qPCR in distal colonic homogenates; colonic sections were subjected to immunofluorescence for Muc2 (red). Scale bar = 150 µm. **i** In wild-type (C57BL/6 J) mice subjected to 3% DSS for 4 days followed by 3 days of plain water, expression of *Spry2* was analyzed in colonic homogenates by qPCR and **j** IL-33 levels were measured in epithelial scraping at 0, 2, 4, and 7 days by ELISA. *p < 0.05 versus day 0. **k** Goblet and tuft cell markers were analyzed in colonic homogenates by qPCR. **p < 0.01; ****p < 0.0001 versus day 0. In figure, data are presented as mean ± SEM. Analyzed by one-way ANOVA with Bonferroni post hoc test or two-sided *t* test. Number of mice analyzed, from five independent litters, in each panel as follows: **a** 11 WT, 16 KO; **b** 8 per group; **c** 4 FF CON, 3 KO CON, 4 FF DSS, 6 KO DSS; **d** 14 FF, 19 KO; **e** 9 FF, 14 KO; **f** 11 FF, 12 KO; **g** *Lgr5* 9 FF, 14 KO; *Lrig1* and *Bmi1* 6 FF, 11 KO; **h** 9 FF, 14 KO; and **i**–**k** *n* = 5 per time point.

Tuft and goblet cells play important roles in protecting the colonic epithelium from damage, as demonstrated by spontaneous colitis in *Muc2*^*−/−*^ mice^4,39^ and increased susceptibility to DSS colitis in *Dclk1-*deficient animals^5,40^. Furthermore, IL-33 ameliorates DSS colitis^41,42^. This suggests that Spry2^IEKO^ mice, which have increased tuft and goblet cell numbers and elevated IL-33, might be protected from experimental colitis. To test this hypothesis, we subjected Spry2^IEKO^ mice and control littermates to acute DSS (3% in drinking water for 4 days, followed by 3 days chase on standard water). Sprouty2 deletion protected mice from weight loss (Fig. 7a), led to improved histopathology scores (Fig. 7b), reduced fecal lipocalin-2 (Fig. 7c), reduced colon shortening (Fig. 7d), and lowered inflammatory cytokine expression (Fig. 7e) following DSS. Apoptosis as measured by TUNEL stain was also significantly reduced in Spry2^IEKO^ colons (Fig. 7f). Notably, expression of the goblet cell marker Muc2 was elevated following DSS in the Spry2^IEKO^ distal colons compared to wild type (Fig. 7h). Furthermore, since Lgr5+ stem cells can be lost in acute inflammatory injury, we measured stem cell markers in distal colon by qPCR, and found that expression of *Lgr5* and *Lrig1* were spared in mice with Sprouty2 deletion in comparison to wild type (Fig. 7g). In contrast, *Bmi1*, which marks reserve stem cells and/or reversion-capable progenitors, was not altered (Fig. 7g).

To understand the temporal kinetics of Sprouty2 regulation by colitis and how it relates to IL-33 levels, we subjected wild-type C57/Bl6 mice to the same model of DSS colitis as above (3% in drinking water for 4 days, followed by 3 days chase on standard water). We found that *Spry2* is lost in the distal colon as early as 2 days after starting DSS (Fig. 7i). Likewise, IL-33 protein (measured in epithelial scrapings) was elevated through day 4, though it returned to baseline by day 7 (Fig. 7j). Expression of goblet cell markers (*Muc2* and *Tff3*) were significantly elevated at day 4. Tuft cell markers (*Dclk1* and *Trpm5*) did not significantly change, though a trend toward increased expression was noted (Fig. 7k). These results suggest that in the presence of severe acute injury, loss of Sprouty2 tracks with increased IL-33 levels in the epithelium. However, either because of incomplete *Spry2* suppression in the tissue or because of other complexities of the colitic tissue environment, these changes are not sustained in wild-type animals.

### Sprouty2 levels are increased in human IBD

**Fig. 8:**
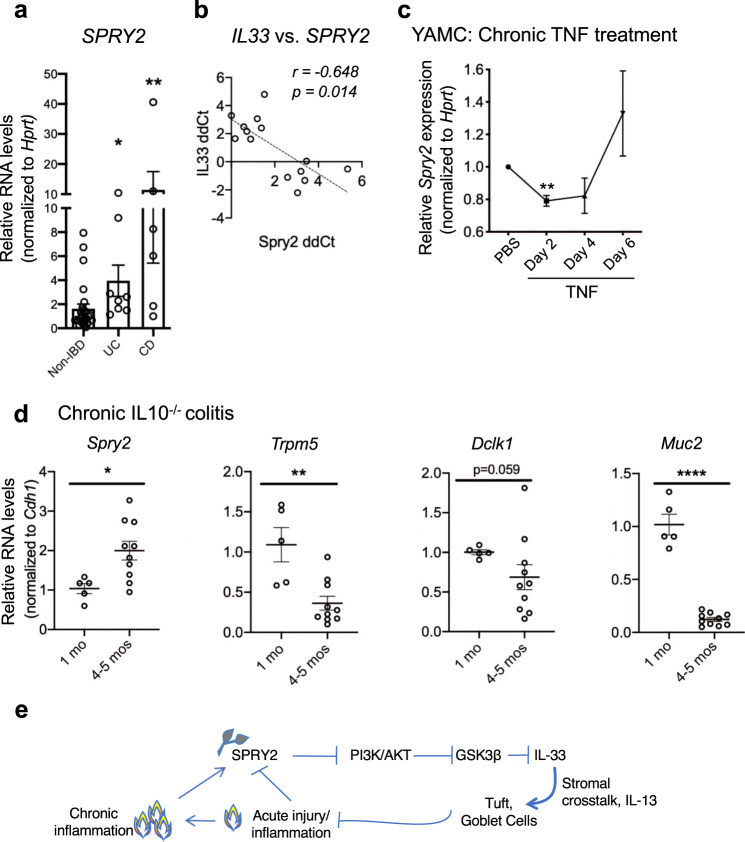
Sprouty2 is elevated in human IBD, and expression levels negatively correlate with tuft cell markers. **a** Samples from patient mucosal biopsies from uninflamed non-IBD (*n* = 27), or inflamed ulcerative colitis (*n* = 8) and Crohn’s disease (*n* = 6), were analyzed for *SPRY2* expression; *p < 0.05; **p < 0.01. Patient characteristics shown in Supplementary Table 1. **b** Correlation of *SPRY2* and *IL33* expression levels from IBD patient biopsies (qPCR, plotted as ddCt values). **c** YAMC cells treated chronically with 100 ng/ml TNF every 2 days for 6 days were measured by qPCR for *Spry2* expression; *n* = 7 independent experiments. **p < 0.01 versus PBS. **d** Distal colonic homogenates from IL10^*−/−*^ mice before onset of colitis (1 month of age; *n* = 5) and post-onset of chronic colitis (4–5 months of age; *n* = 10) were assayed by qPCR for *Spry2* and markers of tuft and goblet cells. *Spry2*, *p = 0.0176; *Trpm5*, **p = 0.0022; *Muc2*, ****p < 0.0001. In figure, data are presented as mean ± SEM. Analyzed by one-way ANOVA with Dunnett post hoc test, Pearson correlation, or two-sided *t* test. **e** Proposed model of Sprouty2 regulation of epithelial IL-33 and secretory cell development in response to colonic inflammation: Acute inflammation represses Sprouty2 expression in the epithelium. This removes basal inhibition on PI3K/Akt, leading to Akt-mediated inhibition of GSK3β, promoting epithelial IL-33 expression. Epithelial-produced IL-33 induces IL-13 in the stroma, which in turn signals back to the epithelium to promote tuft and goblet cell expansion as a compensatory mechanism to limit acute inflammation and promote injury resolution. Loss of this dynamic differentiation mechanism in the setting of chronic inflammation might potentiate disease severity.

Our results in mice suggest that transient Sprouty2 downregulation by inflammation is a compensatory response to early damage signals (e.g., TNF). To consider whether this mechanism might be defective in IBD, we measured *SPRY2* expression in colon endoscopic mucosal biopsies from pediatric IBD patients with active colonic inflammation and non-IBD controls. We found elevated *SPRY2* expression in both ulcerative colitis and Crohn’s disease patients (Fig. 8a and Supplementary Table 1). These data are consistent with findings from Gamo et al. demonstrating elevated *SPRY2* expression in adult IBD patients^43^. Furthermore, in our mucosal biopsy samples, *SPRY2* levels were inversely correlated with *IL33* expression (Fig. 8b). These data suggest that, while acute inflammation can downregulate Sprouty2 to promote IL-33 (Fig. 7), in chronic disease (where *SPRY2* is elevated) this response is lost. To test whether inflammation-induced *Spry2* expression might be lost with chronic stimulation, we challenged mouse colonocytes (YAMC cells) with continuous TNF exposure for 6 days. Acutely, this produced the expected loss of *Spry2*, but by 6 days of exposure this response was lost and *Spry2* levels rebounded (Fig. 8c). This suggests that *Spry2* downregulation by TNF becomes exhausted over time. Consistent with this, in the IL-10^*−/−*^ chronic colitis model, *Spry2* is elevated in colonic homogenates from adult animals with active inflammation versus young (pre-colitic) littermates (Fig. 8d), and these mice express reduced levels of tuft (*Trpm5*) and goblet cell (*Muc2*) markers in the distal colon. A failure to downregulate Sprouty2 in response to early damage signals may be a factor in IBD development (Fig. 8e).

## Discussion

Maintenance of homeostasis and effective responses to injury in the colonic epithelium require a well-regulated balance of different epithelial cell types, including stem, absorptive, and secretory cells. Epithelial-, stromal-, and immune-derived factors direct the development of these cell types^44–48^. However, the precise mechanisms that integrate these signals in the local environment to alter the cellular composition and function of the epithelium are incompletely understood.

In this study, we sought to define the role of Sprouty2 in controlling these processes. Growth factor receptor-initiated signaling, including PI3K and MAPK cascades, is indispensable for intestinal homeostasis^49^ and directs intestinal epithelial cell development and differentiation^9,11,50^. Major receptor tyrosine kinase families, such as the ErbBs and fibroblast growth factor (FGF) receptors, together with their cognate growth factor ligands, activate these cascades. In many cases, administration of the ligands (e.g., NRG1, EGF, and FGF10) alters epithelial cell differentiation and protects against injury or inflammation^10,51^. Sprouty2 can negatively regulate PI3K and MAPK cascades, and is itself regulated by growth factor signals^22,26^, typically induced as a negative feedback inhibitor to keep FGF, EGF, and other signals in check. Here, we show that it is also repressed by acute inflammation both in vitro and in vivo, and thus may play a role in regulating the colonic response to inflammation and injury. As Sprouty2 serves as an integrator and negative regulator of growth factor-induced pathways, it would be predicted that acute downregulation may exert influence on cell development and differentiation, similar to that of activating FGF receptors or ErbBs.

The use of intestinal epithelial-specific Sprouty2 deleted mice (Spry2^IEKO^) allowed us to isolate the effects of Sprouty2 loss from the broader inflammatory milieu of colitis, thus permitting us to directly test its function. Spry2^IEKO^ mice showed increased numbers of secretory tuft and goblet cells, but not enteroendocrine cells or absorptive enterocytes. We speculate this induction may be a compensatory skewing toward cell types that are protective in mouse models of colitis^4,5,52^ and in patients with IBD^53^. Under this scenario, Sprouty2 is constitutively expressed in homeostasis, but is repressed by a danger signal (e.g., TNF) to enable a rapid response to insult. In acute experimental colitis, we observed loss of *Spry2* rapidly during the injury phase (Fig. 7), which correlated with increased IL-33 levels. Subsequently, however, *Spry2* levels remained low while the increased IL-33 was not sustained. We speculate this may be due in part to the aggressive Th1-skewed immune responses following DSS injury. This environment promotes a complex and evolving set of signals that appear to eventually overcome Sprouty2’s effects on IL-33 and IL-33’s role in secretory cell differentiation. Future work with a model where Sprouty2 is constitutively expressed during inflammation will allow us to address the important question of whether failure to downregulate contributes to worsened disease.

To identify downstream mediators driving the effect of Sprouty2 loss in unchallenged mice, we screened for factors known to skew cells along the secretory lineage. This led to the finding that Sprouty2 deletion induces IL-33 production in colonic epithelial cells. IL-33 is expressed by epithelial, stromal, and immune cells, though in homeostasis epithelial expression is normally low^54,55^. IL-33 regulation has predominantly been studied in immune cells^56^ and control of its expression in the intestinal epithelial compartment is not well understood. It has documented roles as a nuclear alarmin and an intracellular regulator of gene function, as well as potentially serving as a traditional secreted cytokine^57^. We found that Sprouty2-regulated IL-33 (Fig. 3) initiated an epithelial–stromal circuit (Fig. 4), and was enriched in media from Sprouty2-null cultured colonic epithelia (Fig. 3), suggesting that it is the secreted IL-33 form functioning here. Furthermore, *Il33* was constitutively elevated in these mice and in Sprouty2-null colonoids, despite no injury markers (epithelial apoptosis or crypt shortening) at baseline (Fig. 2), suggesting that *Spry2* downregulation is a compensatory, downstream response to injury rather than a contributor to it.

Interestingly, while colons from Spry2^IEKO^ mice had a robust phenotype, this was not the case in the small intestine. The underlying mechanism is not entirely clear, but we speculate that the significantly lower baseline Sprouty2 levels in the small intestine (versus colon; Fig. 1) may already be past the signaling threshold to trigger the changes seen in the Spry2^IEKO^ colon. Alternatively, additional regulatory mechanisms or compensation may be active in the small intestine. Future studies will address possible roles of Sprouty2 in the small intestine.

The role of IL-33 in colonic disease is controversial, as some studies suggest it is pathogenic^58,59^ while others have found it is associated with improved outcomes in colitis^41,42^. These different results likely depend on the cellular source of IL-33 and timing of when the cytokine is expressed in the course of disease^60^. Our data suggest that when IL-33 is expressed in the epithelium prior to the onset of disease, it conditions the epithelium against future insult (such as the chemical damage in DSS colitis). Using colonoids from Spry2^IEKO^ and littermate controls, we show that epithelial loss of Sprouty2 directly regulates epithelial IL-33 expression and its impact on secretory cell differentiation is due to extra-epithelial interactions of the cytokine. In addition to promoting intestinal secretory differentiation, IL-33 can induce colonic epithelial wound healing via mir-320 induction^41^. Thus Sprouty2 regulation of IL-33 would be expected to have epithelial-intrinsic pro-repair effects in addition to the protective pro-secretory priming. Indeed, we find that siRNA knockdown of *Spry2* in YAMC cells accelerates restitution in a scratch wound assay (Supplementary Fig. 2), consistent with suppression of restitution in IEC-6 cells by Sprouty2 overexpression^61^. Though this current study is focused on the epithelial–mesenchymal crosstalk effects on secretory differentiation stemming from Sprouty2 deletion, in future studies it will be important to also understand the cell-intrinsic changes brought about by Sprouty2 presence or absence.

While elevated epithelial IL-33 was maintained in Spry2^IEKO^ colonoids following passage, tuft and goblet cell lineage markers were not. Furthermore, exogenous IL-33 did not induce tuft or goblet cell markers. This suggests IL-33 does not promote secretory differentiation by direct epithelial-intrinsic signaling, but rather requires interaction with stromal or recruited cells. Recent work has shown that IL-33 can activate innate lymphoid cells to produce IL-13 (refs. ^57,62^). Waddell et al. demonstrated that this cytokine then reciprocally acts on the intestinal epithelium to promote goblet cell expansion^33^. Consistent with this, we found elevated numbers of IL-13^+^ cells in Spry2^IEKO^ colons; furthermore, IL-13 but not IL-33 induced tuft and goblet cell markers in wild-type colonoids. Importantly, we also confirmed a requirement for IL-13 by treating IL-13 KO and WT mice with IL-33, showing induction of tuft and goblet markers only in WT mice.

Given the induction in IL-33 and tuft and goblet cells in Spry2^IEKO^ mice, it was surprising to not see a coordinate elevation in enteroendocrine cells on the same secretory lineage. Previous work in an IL-33 overexpression model suggested IL-33 mediated elevations in secretory populations included enteroendocrine cells^63^. However the lack of such induction in our model suggests additional variables including environmental factors (e.g., mouse genetics and microbiota), or Sprouty2 signaling modulation may play a role in specific differentiation of cell types in the secretory lineage.

In this study, we have detailed a Sprouty2-regulated intracellular signaling pathway in the colonic epithelium that influences secretory differentiation. Spry2^IEKO^ mice had elevated levels of Akt activation and phosphorylation of the inhibitory serine 9 on GSK3β. Akt mediates this phosphorylation site^38^. This suggests a model where TNF-induced loss of Sprouty2 releases baseline inhibition on Akt signaling, allowing for serine 9 phosphorylation on GSK3β and induction of IL-33. Akt activation by Sprouty2 loss may result from removal of direct inhibition of the PI3K cascade, as Sprouty2 can bind and inactivate components of this pathway^24,25^. Alternatively, gene set enrichment analysis of our RNA-sequencing dataset indicates an upregulation of K-Ras signaling in Spry2^IEKO^ colons (Fig. 6), which could also lead to increased signaling through PI3K-Akt. Future molecular studies are necessary to determine whether these effects encompass any direct actions of Sprouty2 on GSKβ, or strictly are a result of regulating intermediates, such as PI3K. In either case, for IL-33 induction, the pathway converges on GSK3β as demonstrated by the effects of CHIR99021 and LY294002 on colon cells (Fig. 6). Each inhibitor alone regulates *Il33* expression; however, the combined result (attenuation of CHIR99021’s effect by PI3K blockade) is likely due to the mechanism of GSK3β regulation, which involves inhibition by phosphorylation on Ser9 by Akt. Since CHIR99021 serves as an ATP binding competitor for GSK3β rather than a downstream interrupter of signaling, phospho-Ser9 and CHIR99021 should cooperate or synergize to yield full inhibition. Thus, we hypothesize that full inactivation of GSK3β requires Akt blockade and is not completely achieved with CHIR99021 alone at concentrations that are specific for GSK3β. Therefore, only limited (but detectable) *Il33* induction occurs with CHIR99021 when Akt is off.

Our results might indicate a role for Wnt signaling in the control of colonic IL-33, though it should be noted that GSK3β could work through other targets, and thus a Wnt connection will have to be formally tested in future studies. GSK3β and Wnt signaling are known to influence the balance of secretory cells in the intestine^64^, and our work reveals Sprouty2 as an upstream regulator controlled by inflammation and links this pathway to induction of IL-33 (Fig. 6). Previous reports have found that IL-33 influences Akt signaling^65^. However, the impact of PI3K/Akt in control of IL-33 in intestinal tissues has not previously been tested. In accord with Ivanov et al. showing that Akt promotes IL-33 signaling in human fibroblasts^66^, we found that inhibition of the PI3K/Akt cascade repressed basal IL-33 levels from the colonic epithelium. We cannot formally exclude the possibility that altered GSK3β signaling in Spry2^IEKO^ mice also provides feedback to Akt to heighten IL-33 production, but at minimum our in vitro studies do show that IL-33 is regulated by PI3K.

The epithelial switches regulating the IL-33 production in the colonic epithelium, both at homeostasis and in the context of inflammation, are not well understood. Here, we have identified Sprouty2 as an integrator of extracellular stimuli (i.e., TNF) that modulates downstream signaling pathways controlling epithelial IL-33, and thus tuft and goblet cell specification. Tuft cells regulate an inflammatory circuit when activated by parasitic infection^1^, and both tuft and goblet cells protect against colitis in mice^4,5,40^. Furthermore, when these cell types are genetically targeted in mice, experimental colitis is exacerbated. Together these observations suggest that the Sprouty2 circuit described here might be compromised in IBD, a possibility supported by our data showing a significant increase in *SPRY2* in colonic specimens from IBD patients. As some IBD patients with elevated type 2 immune response signatures (such as IL-13) have better outcomes^53^, an important next step will be to understand if Sprouty2 expression or its regulation by TNF is a valid biomarker for IBD severity.

Crohn’s disease and ulcerative colitis are in broad terms characterized by inappropriate or imbalanced activation of Th1 or Th2 immune responses. Prior work has suggested that skewing the immune system through early exposure to parasitic infection can alter the immune tone of the intestine and promote an epithelial composition that may be protective against IBD development^67–69^. Our findings suggest that priming an epithelium to an enhanced secretory tone (by deletion of Sprouty2) limits colitis severity. Interestingly however, we did not detect large scale activation of Th1, Th2, or Th17 immunity in the colons of Sprouty2 mice at baseline (Fig. 5). These findings suggest that, prior to insult, the epithelial–mesenchymal loop regulated by Sprouty2 remodels secretory differentiation without substantively altering immune function beyond ILC2s. It is intriguing to speculate that loss of Sprouty2 may shape the immune response once injury is onset, but due to the fact that elevated tuft and goblet cells are observed prior to injury, and would be predicted to suppress damage, we are unable to dissect primary effects on evolving immune responses versus secondary effects of epithelial-based protection under the current model.

Elevated Sprouty2 in IBD was an unexpected finding, as mucosal biopsies from ulcerative colitis patients (and to a lesser extent Crohn’s disease) can in some studies express elevated IL-33 (ref. ^70^). However, most biopsy samples contain epithelial, mesenchymal, and immune cells, making determination of the IL-33 source in these studies unclear, whereas Sprouty2 is primarily epithelial. Furthermore, the clinical sample set used here was small. Despite this, we observed a significant negative correlation between *SPRY2* and *IL33* levels in mucosal IBD biopsies. As IL-33 may be protective in human IBD, expanded studies correlating Sprouty2, IL-33, and colitis outcomes will be important to pursue to determine whether *SPRY2* acts as a biomarker for disease severity. Longitudinal studies to correlate *SPRY2* expression with tuft and goblet cell markers, and IBD outcomes will be an important step to test our proposed model in patients. It should be noted that these findings are only correlative, and the actual impact of IL-33 on colonic regulation depends on a number of variables. The source, processing (mature versus full-length forms), and localization of IL-33 within the colon likely play key roles in determining the outcome of its effects on tuft and goblet cell development. Specifically in this study, we have found that elevated epithelial IL-33 appears to be protective by directing an epithelial–mesenchymal signaling loop that expands tuft and goblet populations.

While it might seem surprising that *SPRY2* levels are elevated in IBD when they are suppressed in DSS colitis, our data with multiple models suggest that inhibition of Sprouty2 is lost in chronic inflammation. In acute settings, regardless of organism (TNF treatment of either human or mouse epithelia, DSS colitis in the mouse), Sprouty2 levels are suppressed (Figs. 1 and 7). In contrast, in chronic settings (long-term TNF exposure in vitro, IL-10 null mice, and human IBD), this suppression is lost (Fig. 8). This suggests that the apparent discrepancy in DSS colitis versus human IBD is not a species issue (for example, cells from both mice and humans downregulate Sprouty2 in response to TNF), but rather a question of acute signals that are exhausted over time. However, we do not know whether distinct signaling outcomes occur by altered Sprouty2 expression in the setting of chronic inflammation where competing factors may influence cytokine response and secretory cell differentiation. We cannot exclude the possibility that the complex immune environment in colitis may also activate adaptive immune responses that promote Sprouty2 expression, but we were not able to identify such factors in the current study.

In summary, we have demonstrated that Sprouty2 is an inflammation-responsive protein in the colonic epithelium that maintains a cytokine circuit to control tuft and goblet cell numbers. This represents a way in which the epithelium regulates Akt and GSK3β signaling to produce IL-33, and thus alter the composition of the epithelial barrier in a manner that attenuates colonic inflammation.

## Methods

### Study approval

All animal use and experiments received ethical approval, and were monitored by the Children’s Hospital Los Angeles Institutional Animal Care and Use Committee (Animal Welfare Assurance #A3276-01) or the Cincinnati Children’s Hospital Medical Center Institutional Animal Care and Use Committee (Animal Welfare Assurance #A3108-01). All studies complied with relevant ethical regulations for animal testing and research. Mice were housed under standard conditions with ad libitum water and chow access in the AAALAC-accredited animal care facilities at Children’s Hospital Los Angeles or Cincinnati Children’s Hospital Medical Center. Mice were exposed to a half-day light cycle and temperature and humidity-controlled environment. Human tissue was collected after receiving ethical approval for studies and written informed consent was obtained from patients’ parent or legal guardian, under approved Institutional Review Board CCI-13-00287 and CCI-09-00093 at Children’s Hospital Los Angeles. Human studies were conducted in accordance with the criteria set by the Declaration of Helsinki.

### Animal experiments

C57Bl/6 mice were obtained from Jackson Laboratory and used at age 8–10 weeks for experiments. Both male and female mice were used. Mice with an intestinal epithelial-specific deletion of Sprouty2 (VillinCre;Spry2^flox/flox^) were generated by crossing VillinCre (9 kb villin promoter construct) animals^71^ with mice harboring loxP-flanked Spry2 (Spry2^flox/flox^). VillinCre;Spry2^flox/flox^ and Spry2^flox/flox^ littermate controls aged 8–10 weeks were used for experiments. IL-13^*−/−*^ mice and BALB/c controls aged 6–12 weeks were given daily i.p. injections of 0.4 µg rIL-33 for 4 days^33^. For acute colitis, mice were given 3% (w/v) DSS in drinking water for 4 days, followed by 3 days of plain drinking water. Analysis of colonic homogenates, epithelial scrapings, or epithelial peelings are described in figure legends, as appropriate.

### Real-time PCR

RNA from cells and tissue was collected using on-column RNA isolation and purification (OMEGA Biotek), and cDNA generated with a high-capacity cDNA reverse transcriptase kit (Applied Biosystems, 4368814). Quantitative analysis of expression was performed using TaqMan assays (Supplementary Table 2) on an Applied Biosystems StepOne thermocycler. Fold change was calculated using the 2^−ΔΔCt^ method. Results are expressed as average fold change in gene expression relative to control or nontreatment group using *Hprt* or *Cdh1* as the reference gene, as appropriate.

### RNAScope in situ hybridization

Distal colonic sections were probed using RNAScope probes Mm-Spry2 (Advanced Cell Diagnostics, #425061) or Mm-Il13 (Advanced Cell Diagnostics, #312291), using the RNAScope 2.5 HD Detection system (Advanced Cell Diagnostics, #322310) according to manufacturer-provided protocol.

### Western blotting and proteome profiler

Protein lysates from cells and tissue were collected and lysed in RIPA buffer with Halt Protease inhibitor cocktail (Thermo Scientific, #1861278), and phosphatase inhibitor cocktails 2 and 3 (Sigma, P5726 and P0044)^72^. Protein concentration was determined by DC protein assay (Bio-Rad, #500). For proteome profiling, samples were analyzed according to manufacturer instructions using the Phospho-MAPK Array (R&D Systems, #893909). For western blots, 30 μg protein/condition were separated by SDS–PAGE (Thermo Scientific, NW0412A) and transferred to nitrocellulose membrane. Membranes were blocked with 5% milk and probed with 1:1000 Sprouty2 (Sigma, #AV50523) overnight at 4 °C, 1:1000 total and phospho-GSK3beta (S9; Cell Signaling, #12456 and #5558, respectively), 1:1000 total and phospho-Akt (S473; Cell Signaling, #2920 and #4060, respectively), or 1:10,000 mouse anti-Actin (Sigma, A1978) for 1 h at room temperature, followed by 1:20,000 IRDye-conjugated donkey anti-rabbit (LI-COR, #926-68023) and donkey anti-mouse (LI-COR, #926-32212) for 1 h at room temperature and quantification on an Odyssey imager (LI-COR) with Image Studio 4.0. Original scans are included in the source data file.

### ELISA

Fecal lipocalin-2 levels in distal colon fecal contents were analyzed using Mouse Lipocalin-2 DuoSet ELISA (R&D Systems, DY1857) according to manufacturer-provided instructions. IL-13 and IL-33 levels in distal colonic epithelial scrapings or IL-33 in conditioned media from cultures of peeled distal colonic epithelium were analyzed using Mouse IL-13 DuoSet ELISA (R&D Systems, DY413) or Mouse IL-33 DuoSet ELISA (R&D Systems, DY3626) according to manufacturer instructions.

### Cell lines and colonoids

For in vitro experiments, immortalized young adult mouse colon (YAMC) cells, immortalized rat small intestinal cells (IEC-6), and human colon cancer cells (HT-29) were grown to 90% confluency before use in experiments. Colonoids were generated and passaged from mouse colon and human colon using previously established protocols as follows^9,73,74^. Crypts were isolated by incubation of tissue in chelation buffer (2 mM EDTA and 43.4 mM sucrose in D-PBS) followed by shaking. Crypts were embedded in Matrigel (BD Biosciences), then overlaid with growth media for mouse (advanced DMEM/F12 [Invitrogen, #12634-010] supplemented with 2 mM GlutaMax [Invitrogen, 25030-081], 10 mM HEPES [Sigma, H0887], 1× Pen–Strep [Invitrogen, 15140-122], 1× N2 supplement [Invitrogen, #17502048], 1× B27 supplement [Invitrogen, 17504044], 10% R‐spondin conditioned media [from modified HEK-293T cell line generated by Dr. Jeffrey Whitsett at Cincinnati Children’s Hospital Medical Center], 100 ng/ml mNoggin [PeproTech, 250-38], 50 ng/ml mEGF [PeproTech, 315-09], and 3 µM CHIR99021 [PeproTech, 2520691]) or human (advanced DMEM/F12 supplemented with 2 mM GlutaMax, 10 mM HEPES, 1× Pen–Strep, 1× N2 supplement, 1× B27 supplement, 10% R‐spondin CM, 100 ng/ml hNoggin [Peprotech, #120-10X], 50 ng/ml hEGF [Invitrogen, #PHG0311], 500 nM LY-2157299 [AxonMedChem, #1491], 10 µM SB-202190 [Sigma, #S7067], 50 U/ml Gentamycin [Invitrogen, 15710064], and 0.25 µg/ml amphotericin B [Invitrogen, 109434]). Cultures were passaged every 7–10 days. Cultures were starved in basal medium (advanced DMEM/F12 supplemented with 2 mM GlutaMax, 10 mM HEPES, 1× Pen–Strep, 1× N2 supplement, and 1× B27 supplement) then treated with the indicated concentrations of murine TNF (Peprotech, #315-01), human TNF (Peprotech, #300-01), mouse IL-33 (Peprotech, #210-33), or mouse IL-13 (Peprotech, #210-13) for the time specified before collection.

### Immunofluorescence staining

Distal colon sections (5 µm), from tissue fixed with 4% formaldehyde overnight and paraffin embedded, were dewaxed and blocked with 10% goat serum for 1 h at room temperature. This was followed by incubation with primary antibody against DCLK1 (Abgent, #AP7219B, 1:100), ChgA (ImmunoStar, #20085, 1:200), Muc2 (Santa Cruz, sc-15334, 1:1000), Gata3 (Cell Signal, #5852, 1:200), Ki67 (Thermofisher, #RM-9106, 1:200), or E-cadherin (BD Biosciences, #610181, 1:500) overnight at 4 °C. Cells were washed and incubated with secondary anti-mouse Alexa Fluor-488 (Life Technologies, #A11029, 1:1000) or anti-rabbit Alexa Fluor-546 (Life Technologies, #A11035, 1:1000) for 1 h at room temperature followed by mounting with Vectashield mounting media including DAPI (Vector Labs, H-1500). TUNEL stain (Roche, #11684795910) was performed according to manufacturer instructions^75^ on distal colonic sections from DSS-treated mice. Imaging was performed on a ZEISS 710 confocal system using Zen 2009 software.

### Human tissue

Colonic tissues from endoscopic biopsies (for *SPRY2* expression analysis) or gross surgical specimens (for colonoid generation) were obtained from pediatric patients either with or without IBD at Children’s Hospital Los Angeles. Samples were placed immediately in RNALater (Thermo Fisher Scientific, AM7021) or used for colonoid generation.

### RNA sequencing and analysis

RNA isolated from distal colonic homogenates (from six Sprouty2^FF^ and six Sprouty2^IEKO^ littermates) were barcoded using Illumina index primers and sequenced on a NextSeq machine to obtain single-ended reads of 75 bp length to a depth of ~10 M reads/sample by SeqMatic LLC (San Francisco, CA). FASTQ files were pseudoaligned using kallisto^76^ to the mouse transcriptome to obtain transcript per million abundance estimates. To compare pathway enrichment, transcript abundances were summed to obtain a per-gene expression value, which was then input into GSEA 4.1.0 (ref. ^77^). Enrichment of the KRAS pathway was identified using the Hallmark database of targets^78^. Specific analysis for enrichment of ILC2 markers was performed using the GSEA algorithm against a previously identified ILC2 gene set profile enriched in intestinal tissue^31^ For immune cell estimation in colonic tissue, gene expression data (with HUGO gene symbols) were submitted to the CIBERSORT portal (http://cibersort.stanford.edu) and processed, using the LM22 signature gene file at 100 permutations^34^. Results were standardized as relative abundance of the total predicted immune cell populations.

### Statistics

Statistical analyses and plots were generated using Prism 6 (GraphPad Software). Mean ± SEM is depicted in dot and bar graphs. All data points (dots) on plots represent biological replicates. Student’s two-sided *t* test or one-way ANOVA with Tukey, Dunnett, or Bonferroni post hoc test to correct for multiple comparisons were used to determine statistical differences, as indicated in figure legends. Statistical significance was assigned to *p* < 0.05, and indicated in figure legends.

### Reporting summary

Further information on research design is available in the Nature Research Reporting Summary linked to this article.

## Supplementary information

Supplementary Information

Reporting Summary

## Data Availability

RNA-sequencing data for the results presented in Figs. 4–6 are freely and publicly available through the NCBI Gene Expression Omnibus; the accession number is GSE160854. The authors declare that all other data supporting the findings of this study are available from the corresponding author upon reasonable request. Source data are provided with this paper.

## Source data

Source Data
