# Supplementary material for: Sprouty2 limits intestinal tuft and goblet cell numbers through GSK3β-mediated restriction of epithelial IL-33
Source: Nat Commun. 2021 Feb 5;12:836. doi: 10.1038/s41467-021-21113-7 (PMC7864916; doi:10.1038/s41467-021-21113-7)
Supplement: Supplementary file 1 — Supplementary Information [file 41467_2021_21113_MOESM1_ESM.pdf]

Supplementary Figure 1. *Spry2 k*nockdown in cultured mouse colonocytes enhances wound restitution.

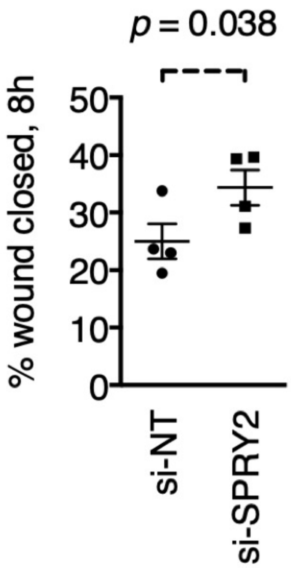

Mouse young adult mouse colon epithelial cells (YAMC cell line) were transfected with non-targeting (NT) siRNA or siRNA targeting *Spry2* (Dharmacon). 48 h later, confluent monolayers were wounded with a rotating silicone tip, and wound closure over time was tracked by time-lapse photography. n=4 independent experiments. Data are presented as mean ± SEM. Analyzed by two-sided t-test.

Supplementary Table 1

**Patients**	**Avg. age (years)**	**Male (%)**	**Female (%)**
Non-IBD	12.3 ± 5.049	51.9	48.1
UC	13.1 ± 4.998	37.5	62.5
CD	16.7 ± 2.875	66.7	33.3

Supplementary Table 2

**Taqman qPCR probes used in paper**			
**Manufacturer**	**Gene name**	**Species**	**Probe ID**
Invitrogen	*Aqp8*	Mouse	Mm00431846_m1
Invitrogen	*Bmi1*	Mouse	Mm03053308_g1
Invitrogen	*Car2*	Mouse	Mm00501576_m1
Invitrogen	*Cdh1*	Mouse	Mm01247357_m1
Invitrogen	*Chga*	Mouse	Mm00514341_m1
Invitrogen	*Cxcl2*	Mouse	Mm00436450_m1
Invitrogen	*Cxcl2*	Rat	Rn00586403_m1
Invitrogen	*Dclk1*	Mouse	Mm00444950_m1
Invitrogen	*Hprt*	Mouse	Mm03024075_m1
Invitrogen	*Hprt*	Rat	Rn01527840_m1
Invitrogen	*HPRT*	Human	Hs02800695_m1
Invitrogen	*Il10*	Mouse	Mm01288386_m1
Invitrogen	*Il1b*	Mouse	Mm00434228_m1
Invitrogen	*Il25*	Mouse	Mm00499822_m1
Invitrogen	*Il33*	Mouse	Mm00505403_m1
Invitrogen	*Il33*	Rat	Rn01759835_m1
Invitrogen	*IL33*	Human	Hs04931857_m1
Invitrogen	*Lgr5*	Mouse	Mm00438890_m1
Invitrogen	*Lrig1*	Mouse	Mm00456116_m1
Invitrogen	*Muc2*	Mouse	Mm01276696_m1
Invitrogen	*Pou2f3*	Mouse	Mm00478293_m1
Invitrogen	*Spry2*	Mouse	Mm00442344_m1
Invitrogen	*SPRY2*	Human	Hs01921749_s1
Invitrogen	*Tff3*	Mouse	Mm00495590_m1
Invitrogen	*Tgfb*	Mouse	Mm01178820_m1
Invitrogen	*Tnf*	Mouse	Mm00443258_m1
Invitrogen	*Trpm5*	Mouse	Mm01129032_m1
