# Supplementary material for: Sprouty2 limits intestinal tuft and goblet cell numbers through GSK3β-mediated restriction of epithelial IL-33
Source: Nat Commun. 2021 Feb 5;12:836. doi: 10.1038/s41467-021-21113-7 (PMC7864916; doi:10.1038/s41467-021-21113-7)
Supplement: Supplementary file 2 — Reporting Summary [file 41467_2021_21113_MOESM2_ESM.pdf]

nature research

Corresponding author(s): Mark Frey

Last updated by author(s): Dec 1, 2020

Reporting Summary

Nature Research wishes to improve the reproducibility of the work that we publish. This form provides structure for consistency and transparency in reporting. For further information on Nature Research policies, see our Editorial Policies and the Editorial Policy Checklist.

Statistics

For all statistical analyses, confirm that the following items are present in the figure legend, table legend, main text, or Methods section.

n/a	Confirmed	
☐	☒	The exact sample size (n) for each experimental group/condition, given as a discrete number and unit of measurement
☐	☒	A statement on whether measurements were taken from distinct samples or whether the same sample was measured repeatedly
☐	☒	The statistical test(s) used AND whether they are one- or two-sided<br>*Only common tests should be described solely by name; describe more complex techniques in the Methods section.*
☐	☒	A description of all covariates tested
☐	☒	A description of any assumptions or corrections, such as tests of normality and adjustment for multiple comparisons
☐	☒	A full description of the statistical parameters including central tendency (e.g. means) or other basic estimates (e.g. regression coefficient) AND variation (e.g. standard deviation) or associated estimates of uncertainty (e.g. confidence intervals)
☐	☒	For null hypothesis testing, the test statistic (e.g. F, t, r) with confidence intervals, effect sizes, degrees of freedom and P value noted<br>*Give P values as exact values whenever suitable.*
☒	☐	For Bayesian analysis, information on the choice of priors and Markov chain Monte Carlo settings
☒	☐	For hierarchical and complex designs, identification of the appropriate level for tests and full reporting of outcomes
☐	☒	Estimates of effect sizes (e.g. Cohen's d, Pearson's r), indicating how they were calculated

Our web collection on statistics for biologists contains articles on many of the points above.

Software and code

Policy information about availability of computer code

Data collection	ABI StepOne version 2.3, Zeiss ZEN 2009, Odyssey, LICOR Image Studio 4.0
Data analysis	Graphpad Prism 6, ABI StepOne version 2.3, Zeiss ZEN 2009, Odyssey LICOR Image Studio 4.0, GSEA 4.1.0, CIBERSORT v1, kallisto v0.44.0

For manuscripts utilizing custom algorithms or software that are central to the research but not yet described in published literature, software must be made available to editors and reviewers. We strongly encourage code deposition in a community repository (e.g. GitHub). See the Nature Research guidelines for submitting code & software for further information.

Data

Policy information about availability of data

All manuscripts must include a data availability statement. This statement should provide the following information, where applicable:

- Accession codes, unique identifiers, or web links for publicly available datasets
- A list of figures that have associated raw data
- A description of any restrictions on data availability

RNA sequencing data supporting the results presented in Figures 4, 5, and 6 are freely and publicly available through the NCBI Gene Expression Omnibus (GSE160854). Hallmark dataset used in Figure 6 is freely available from reference 78 in manuscript. ILC2 expression dataset is available from reference 31 in manuscript. The LM22 dataset used in CIBERSORT is available at the CIBERSORT portal (http://cibersort.stanford.edu). Source data for all figures are included in supplementary materials.

Field-specific reporting

Please select the one below that is the best fit for your research. If you are not sure, read the appropriate sections before making your selection.

- [x] Life sciences
- [ ] Behavioural & social sciences
- [ ] Ecological, evolutionary & environmental sciences

For a reference copy of the document with all sections, see nature.com/documents/nr-reporting-summary-flat.pdf

Life sciences study design

All studies must disclose on these points even when the disclosure is negative.

Sample size	Cohort size defined based on biological variation from pilot experiments & designed to give >80% power to detect 2-fold changes in expression patterns.
Data exclusions	One data point in Figure 6F, Cxcl2 panel, was excluded after detection by ROUT analysis (with Q set to 0.1% to remove only definitive outliers).
Replication	In vivo experiments were replicated across samples derived from at least 3 independent litters; in vitro experiments were replicated across multiple cell lines and primary tissue through at least 3 independent experiments. Replication of in vivo and in vitro experiments between litters and multiple cell lines was successful and results are included in the manuscript.
Randomization	Experimental groups (KO vs WT) were naturally randomized due to breeding scheme with sex and genotype equally distributed due to use of littermate controls
Blinding	Investigators were blinded to experimental groups for collection of samples and sample analysis

Reporting for specific materials, systems and methods

We require information from authors about some types of materials, experimental systems and methods used in many studies. Here, indicate whether each material, system or method listed is relevant to your study. If you are not sure if a list item applies to your research, read the appropriate section before selecting a response.

Materials & experimental systems

n/a	Involved in the study	
[ ]	[x]	Antibodies
[ ]	[x]	Eukaryotic cell lines
[x]	[ ]	Palaeontology and archaeology
[ ]	[x]	Animals and other organisms
[ ]	[x]	Human research participants
[x]	[ ]	Clinical data
[x]	[ ]	Dual use research of concern

Methods

n/a	Involved in the study	
[x]	[ ]	ChIP-seq
[x]	[ ]	Flow cytometry
[x]	[ ]	MRI-based neuroimaging

Antibodies

Antibodies used	DCLK1 (Abgent, #AP7219B), ChgA (ImmunoStar, #20085), Muc2 (Santa Cruz, sc-15334), E-cadherin (BDBiosciences, #610181), Sprouty2 (Sigma, #AV50523), total and phospho-GSK3beta (S9) (Cell Signaling, #12456, #5558, respectively), total and phospho-Akt (S473) (Cell Signaling, #2920, #4060, respectively), mouse anti-Actin (Sigma, A1978), Gata3 (Cell Signal, #5852), Ki67 (ThermoFisher, RM-9106), goat anti-mouse Alexa Flour-488 (Invitrogen, #A11029), goat anti-rabbit Alexa Flour-546 (Invitrogen, #A11035), IRDye-conjugated donkey anti-rabbit (LI-COR, #926-68023) and IRDye-conjugated donkey anti-mouse (LI-COR, #926-32212).
Validation	Antibodies are well-characterized with documented validation from commercial vendors on applicability to western blot or immunofluorescence as used in the manuscript. Antibodies have previous publications documenting validity in mouse and humans, as used in this manuscript. Manufacturer has validated DCLK1 (Abgent, #AP7219B) reactivity with mouse and its use in intestinal murine tissue for IF in documented in PMID 19879217. Manufacturer has validated ChgA (ImmunoStar, #20085) reactivity in mouse and its use in intestinal murine tissue for IF is documented in PMID 22001866. Manufacturer has validated Muc2 (Santa Cruz, sc-15334) and its use in intestinal murine tissue for IF is documented in 24292484. Manufacturer has validated E-cadherin (BDBiosciences, #610181) reactivity in mouse and its use in IF staining. Manufacturer has validated Spry2 and Actin (Sigma, AV50523, A1978) reactivities in mouse and their use in western blotting. Manufacturer has validated total and phospho-GSK3beta (S9) (Cell Signaling, #12456, #5558, respectively), & total and phospho-Akt (S473) (Cell Signaling, #2920, #4060, respectively) reactivities in mouse and their use in western blotting. Manufacturer has validated Gata3 (Cell Signal, #5852) reactivity in mouse and its use in IF. Ki67 (ThermoFisher, RM-9106) has previously published usage in mouse for immunostain (PMID 18790560).<br><br>Immunofluorescent staining and western blots showed relatively little background noise and proteins were detected at expected kDa sizes on western blots.

Eukaryotic cell lines

Policy information about cell lines

Cell line source(s)	YAMC (young adult mouse colon) cells are an immortalized murine colon cell line and were obtained from the original investigator creating the line (Robert Whitehead). IEC-6 (rat intestinal cells) are an immortalized small intestinal rat cell line and were obtained from ATCC. HT-29 cells (human colon cells) are a cell line derived from human colon adenocarcinoma and were obtained from ATCC.
Authentication	YAMC were used from frozen stocks tested for the presence of the immorto-transgene used to generate the line. IEC-6 and HT-29 cells were validated by ATCC and STR testing.
Mycoplasma contamination	Cell lines tested negative for mycoplasma.
Commonly misidentified lines (See ICLAC register)	No commonly misidentified cell lines were used.

Animals and other organisms

Policy information about studies involving animals; ARRIVE guidelines recommended for reporting animal research

Laboratory animals	Mice; C57Bl/6, Sprouty2-Floxed, VillinCre, IL10KO, BALB/c, and IL-13KO strains were used at an equal male/female mix used at similar ages as specified in the manuscript. Specifically, mice aged 8-10 weeks were used for all in vivo studies except IL10-KO (used at either 1 month of age or 4-5 months of age), and BALB/c and IL-13KO mice (used at 6-12 weeks of age).
Wild animals	Study did not involve wild animals.
Field-collected samples	No field-collected samples were used in the study.
Ethics oversight	All animal use was approved and monitored by the Children's Hospital Los Angeles Institutional Animal Care and Use Committee (Animal Welfare Assurance #A3276-01) or the Cincinnati Children's Hospital Medical Center Institutional Animal Care and Use Committee (Animal Welfare Assurance #A3108-01).

Note that full information on the approval of the study protocol must also be provided in the manuscript.

Human research participants

Policy information about studies involving human research participants

Population characteristics	Non-IBD patients were an average age of 12.3 ± 5.049 (51.9% male/48.1% female)<br>UC patients were an average age of 13.1 ± 4.998 (37.5% male/62.5% female)<br>CD patients were an average age of 16.7 ± 2.875 (66.7% male/33.3% female)
Recruitment	Patients were recruited on routine biopsy.
Ethics oversight	Human tissue was collected after receiving ethical approval for studies and written informed consent was obtained, under approved Institutional Review Board CCI-13-00287 and CCI-09-00093, respectively, at Children's Hospital Los Angeles.

Note that full information on the approval of the study protocol must also be provided in the manuscript.
